# From Personal to Public Health - building Health Data Pipelines

**DOI:** 10.1093/eurpub/ckac129.082

**Published:** 2022-10-25

**Authors:** S Buttigieg

**Affiliations:** 1 EUPHA-DH; 2 Digital Health Malta, Msida, Malta

## Abstract

The creation of future proof infostructures requires a number of efforts on different levels within a health organisation. One important thing that we need to be genuinely realise and grow aware of is that there is an inherent need to move away from Excel Spreadsheets, Access Databases and legacy data tools (including paper) towards interoperable information systems that are focused on high-quality data coordination mechanisms, robust processes and documentation and most of all design of data flows that enable sustainable data collection and analysis. This hands-on session will inform users on the following elements:

– Doing a thorough situation analysis of your current data situation

– Design your health data pipeline - paper and imagination required

– Resources to help you design the ideal infostructure

– Setting your next steps forward

– Being aware of your organisational culture to ensure maximum success

